# Tumor control and survival after postoperative radiotherapy for high-risk oral cavity cancer: A retrospective cohort study

**DOI:** 10.1016/j.ctro.2025.100988

**Published:** 2025-05-30

**Authors:** Pepijn B Bolleurs, Brend P Jonker, Joris BW Elbers, Gerda M Verduijn, Atilla Gül, Aniel Sewnaik, Wilma D Heemsbergen

**Affiliations:** a Department of Radiotherapy, Erasmus MC Cancer Institute, University Medical Center Rotterdam, Rotterdam, the Netherlands; b Erasmus University College, Rotterdam, the Netherlands; c Department of Oral and Maxillofacial Surgery, Erasmus MC Cancer Institute, University Medical Center Rotterdam, Rotterdam, the Netherlands; d Department of Oral and Maxillofacial Surgery and Head and Neck Surgery, Elisabeth-TweeSteden Hospital, Tilburg, the Netherlands; e Department of Otorhinolaryngology and Head and Neck Surgery, Erasmus MC Cancer Institute, University Medical Center Rotterdam, Rotterdam, the Netherlands

**Keywords:** Oral squamous cell carcinoma, Postoperative radiotherapy, Locoregional control, Survival

## Abstract

**Background:**

We evaluated locoregional failure (LRF) and survival after postoperative radiotherapy (PORT) in selected high-risk oral squamous cell carcinoma (OSCC) patients.

**Methods:**

In a retrospective OSCC cohort (n = 219) treated with local (n = 216) and/or regional (n = 87) PORT in 2011–2018, we determined the first location of tumor recurrence or progression, survival, and cause of death. Tumor control and survival was calculated using Kaplan Meier method. Prognostic factors were evaluated in Cox regression models.

**Results:**

Main subsites were oral tongue (34 %), gingiva (32 %), and floor of mouth (27 %). Eight percent also received chemotherapy. Median follow-up was five year for tumor control and eight year for survival. Tumor progression was observed in 47 patients (n = 31 LRF). Nine patients had salvage treatment. Locoregional control was 87 % and 84 % at 2 and 5 years, respectively. Significant prognostic factors for local failure (LF) were T4 stage, bone invasion, and subsite gingiva; 12 of 18 LF concerned a T4 gingiva tumor with bone invasion. For regional failure (RF), pN1 (vs pN0) was prognostic, mainly concerning solitary contralateral RFs. Overall survival was 63 % and 48 % at 5 and 8 year, respectively. Main causes of death (104 events) were the index tumor (n = 42) and a post-treatment second primary tumor (n = 37).

**Conclusion:**

A locoregional control of 84 % was achieved after PORT for high-risk OSCC with only 8 % receiving additional chemotherapy. Overall survival was 48 % at 8 years, with a large proportion of cancer-related deaths related to the index tumor and other subsequent tumor diagnoses. Risk of LF was increased for gingiva tumors with bone invasion. N1 stage was associated with a risk of solitary contralateral RFs in non-irradiated neck areas.

## Highlights

• Tumor control and survival after PORT in a high-risk oral cavity cohort was assessed.
• Locoregional control was 84 % with most local failures in T4 gingiva tumors.
• Regional failures were mainly contralateral in non-targeted neck areas.
• Overall survival was 48 % at 8 years, mainly cancer-related deaths.
• Cancer-related deaths considered index tumors as well as subsequent new tumors.

## Introduction

Globally, oral squamous cell carcinoma (OSCC) is the most frequently diagnosed head & neck malignancy [1]. The primary treatment for OSCC patients is surgery. Post-operative radiotherapy (PORT) plays an important role as adjuvant treatment in case of unfavorable pathological disease characteristics, improving locoregional tumor control and associated disease-specific survival [[2], [3], [4]]. PORT is indicated when pathological risk factors are present [[2], [3], [4], [5], [6]]. PORT to the tumor bed is usually prescribed in case of <1 mm resection margin, or presence of (multiple) minor risk factors: tight (<5 mm) surgical margin, T3-4 stage, perineural invasion, budded growth, or lympho-vascular invasion [[2], [3], [4], [5], [6]]. PORT to the neck is usually prescribed to patients with pN2-3 or patients with extranodal extension (ENE), and only in pN1 cases when high-risk factors are present, although this is still under debate [[7], [8], [9]].

In the past decade, guidelines on patient selection and radiotherapy protocols for PORT have been evolving based on new insights, in order to avoid under- or over-treatment, since PORT is associated with significant toxicity risks impacting quality of life [9,10]. Target delineation in PORT remains challenging due to postsurgical anatomical changes and the absence of international consensus guidelines, resulting in inter-observer variability in treatment planning [11]. A recent review summarized the evidence on PORT target definitions, and recommended consensus guidelines as a result of a multidisciplinary convention, in order to support standardization in PORT treatment planning [11].

In general, locoregional failures in squamous cell head & neck cancer treated with radiotherapy are common, affecting their further prognosis and quality of life [12]. Knowledge concerning patterns of first failure sites and survival after PORT is important for evaluating and improving treatment protocols for selected patients, and to gain more insight into the prognosis [[13], [14], [15]]. We performed a retrospective cohort study in a large series of OSCC PORT patients with pathologic high-risk factors treated at our institute, to investigate outcomes after PORT. The primary goal was to assess locoregional tumor failure rates, overall survival, and the distribution of causes of death. Secondary objectives were to evaluate prognostic factors for local failure, regional failure, and survival.

## Methods and materials

### Study patient selection

**Fig. 1:**
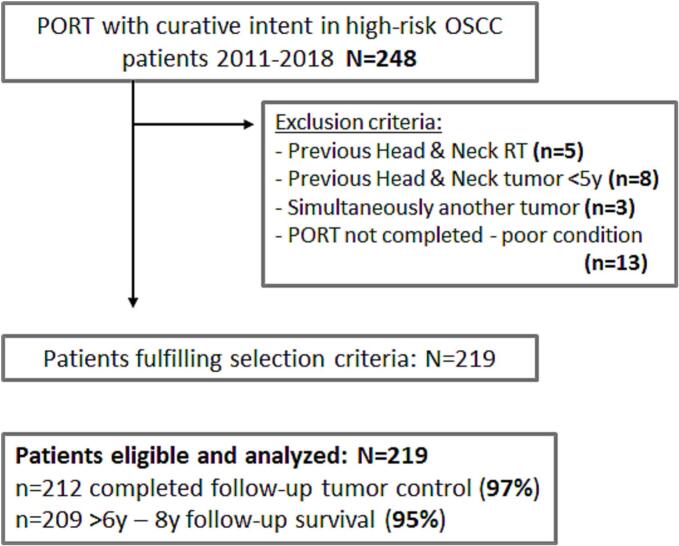
Flow chart of study patient selection according to inclusion and exclusion criteria.

We performed a single-center retrospective cohort study at the Erasmus Medical Cancer Institute, a university-affiliated tertiary hospital. The study protocol was reviewed and approved by the Medical Ethical Committee of the Erasmus Medical Center, Rotterdam, the Netherlands (EMC 2020-0020), and permission for retrospective collection of anonymized clinical and dosimetric data was obtained. We identified all OSCC patients in our radiotherapy planning system who were scheduled for PORT between 2011–2018 with curative intent based on pathological risk features (N = 248). From this initial selection, patients files were further checked for eligibility, and subjects excluded in case they had a history of a previous head and neck cancer < 5 year, previous radiotherapy in the head and neck region, simultaneous treatment of two primary tumors, or did not complete PORT. Following in- and exclusion criteria, N = 219 patients were eligible (Fig. 1**)**.

### Indication for PORT

At the time of treatment of the first patient within this cohort in 2011, PORT criteria were largely based on the Dutch national guidelines from 2004 [16]. PORT within 6 weeks was indicated for high-risk patients. Tumor bed PORT was indicated in case of ≥1 of the following hard criteria: positive surgical margins/<1mm, lymph node metastasis with ENE or multiple lymph node metastases (N2b-N3), or ≥2 of the following soft criteria: T3-T4 tumors, tight surgical margins (>1 mm ≤5 mm), perineural invasion, a budding growth pattern (now termed worst pattern of invasion). Regarding PORT of the neck, the Dutch guidelines from 2004 conclude that with a tumor at least 1 cm away from the midline after an elective lymph node dissection with a pN0-N1 neck (without ENE), PORT for neck areas is not indicated [16]. In the case of pN1 (single ipsilateral lymph node 3 cm or less in greatest dimension) with ENE, the neck was irradiated (in the study cohort the AJCC TNM 7th edition staging for oral cancer was used; in the 8th edition any node with ENE is considered at least N2a or higher). Recent published guidelines from 2018 and 2019 are in agreement with this protocol for neck PORT [4,6]. Lymphovascular space invasion has been added as a soft criterion to the current guidelines for PORT (after 2020). Over time, an additional criterion was added: a lymph node larger than 3 cm (N2a). PORT was omitted in patients for whom only soft criteria were met if there were strong contraindications for PORT. From 2013 onwards, concomitant chemotherapy was indicated for PORT patients aged ≤70 years with surgery margins <1 mm or ENE, based on evidence from studies and updated guidelines [[17], [18], [19]]. Accelerated PORT (with 6 fractions per week, 2 Gy) was prescribed up to November 2014 in all patients (but not in case of chemotherapy + PORT, or schedules with 2.5 Gy fractions, and not in some cases with a poor condition); from that point onwards, the protocol was changed and all patients received 5 fractions per week.

### Surgical procedures

Patients received initial surgery which consisted of resection of the tumor, and in some cases combined with neck dissections (ND) and reconstructions. Unilateral ND of level I-III with a clinical N0 neck was performed in case of a depth of invasion (DOI) of >4 mm for tongue and a DOI of >3 mm for all other tumor subsites. Unilateral elective ND of level I-IV was performed in case of a clinical N1 neck. Contralateral ND of levels I-III was performed in case of tumor crossing the midline.

### Radiotherapy planning and target definitions

For an optimal determination of the clinical target volume (CTV), pre- and postoperative information was assessed, including pre-operative CT and/or MRI of the head and neck, clinical photographs of the primary tumor, ultrasound of cervical lymph nodes, and an orthopantomogram. A thoracic CT was performed if lymph node metastases were present in level IV or if the disease was classified as stage N2b. Additionally, a planning CT scan was obtained between two and six weeks after surgery. For CTV delineation, the post-operative planning CT was fused with the pre-operative CT or MRI. Surgical and pathology reports were also considered.

In some cases with a N0 neck, node levels Ia, Ib, II and III were included at the discretion of the treating physician (6 out of 109 pN0 cases had neck PORT). In case of N + neck, node levels I-IV were included; level V only when indicated. Additionally, higher dose levels were prescribed to node areas with pathological nodes including a local boost to areas with ENE. Bilateral neck RT was indicated when the pre-operative tumor bed extended to or over the midline. Prescribed dose (2 Gy fractions) was: 66 Gy for high-risk areas (positive surgical margins or ENE), 60 Gy for intermediate-risk areas (no positive surgical margins and no ENE) (changed to 56 Gy in 2 Gy fractions from 2016 onwards), and 46–50 Gy for electively irradiated levels. The high-dose CTV margin was 1.0 cm. Patients with unfavorable performance status were offered a schedule with a reduced number of hospital visits (22–24 fractions of 2.5 Gy). The RT technique was intensity-modulated radiotherapy (IMRT).

### Follow up and recurrence identification

Patients were scheduled for multidisciplinary follow-up (oral-maxillofacial surgeon, ear/nose/throat (ENT) physician, radiation oncologist) every 2–4 months during years 1–2 and every 6 months during years 3–5. Tumor progression was typically identified via clinical symptoms, physical examination or imaging, with histopathological confirmation when possible, although some patients declined further testing. Recurrences were discussed and classified in multidisciplinary meetings, and these consensus reports provided the primary data on tumor progression/recurrence for the current study. Radiotherapy planning data were used to determine targeted areas (tumor bed yes/no, ipsilateral, and contralateral neck levels). Three investigators (PB, WH, JE) reviewed all recurrences and classified them as within or outside the PORT target area based on available information. For example, a recurrence in a right level IV lymph node was scored as ‘outside RT field’ if PORT was delivered only to the left neck. Regional failures could be scored with confidence. For local failures, it could only be determined whether the tumor bed was included in the targeted PORT area yes/no; it was not possible to determine the exact in/outfield location of local recurrences with respect to the local tumor bed delineation and dose distribution, since we lacked recurrence imaging for a significant number of patients. Survival data were collected for up to eight years post-treatment, as most patients had hospital visits during this period.

### Statistical analysis

Freedom from tumor progression and survival rates were calculated with the Kaplan-Meier method. Time was calculated from start RT. Censoring was done at reaching another failure endpoint, diagnosis of a secondary malignancy, death, completed follow-up, or at last evaluation date (1-10-2024), whichever came first. Prognostic factors were evaluated in Cox proportional hazards models (evaluated factors in the Results section). In case of categorical variables, the largest subgroup was the reference group. For ordinal variables, the lowest value was the reference. For overall survival, variables were analyzed in a baseline model including age at RT. Analyses were performed using SPSS software (version 26, IBM corporation, Armonk, NY). For multivariable analysis, conditional backward selection was performed (with removal at 0.1 level).

## Results

### General aspects

Baseline characteristics are presented in Table 1. Patients received PORT to either the tumor bed only (n = 132), neck areas only (n = 3), or both (n = 84). Main subgroups of the gingival subsite were the alveolar process (n = 42 mandible, n = 13 maxilla) and the retromolar trigone of the mandible (n = 13). Two patients had a history of OSCC tumor > 5 years ago (treated with local dissection). Distributions of risk factors per tumor subsite (supplementary Table A1) showed that gingiva tumors were mainly T4 (87 %). Median interval between surgery and PORT was 6.1 weeks (range 3–17). Median follow up for tumor control was 5 years.

**Table 1:** Baseline characteristics of the study cohort (n = 219). For TNM staging, the AJCC 7th Edition was used.

Baseline Characteristics	Number of Patients (%)
*Patient Characteristics*	
Mean age at PORT (years)	64.6 (11.3 1SD, range 24–89)
Sex	
Male	137 (62.6)
Female	82 (37.4)
Smoking status at diagnosis	
Never smoker	34 (15.5)
Current smoker	110 (50.2)
Previous smoker	75 (34.2)
Charlson Comorbidity score 0 1 2 ≥3 (maximum 5)	107 (48.9 %) 48 (21.9 %) 37 (16.9 %) 27 (12.3 %)
*Tumor Characteristics*	
T stage pathology	
T1	39 (17.8)
T2	66 (30.1)
T3	19 (8.7)
T4a	91 (41.6)
T4b	4 (1.8)
N stage pathology	
N0	116 (53.0)
N1	33 (15.1)
N2A	4 (1.8)
N2B	46 (21.0)
N2C	16 (7.3)
N3	4 (1.8)
Oral cavity sub-site	
Floor of mouth	60 (27.4)
Oral Tongue	74 (33.8)
Gingiva	71 (32.4)
Buccal mucosa	14 (6.4)
Bone invasion	75 (34.2)
Positive margins	65 (29.7)
Positive margins or surgical margins <1 mm	88 (40.2)
Positive margins or surgical margins <5 mm	180 (82.2)
Budding growth pattern	148 (67.6)
Peri-neural invasion	86 (39.3)
Lymphovascular space invasion	39 (17.8)
Differentiation grade	
Good	12 (5.5)
Moderate	141 (64.4)
Poor	66 (30.1)
*Treatment Characteristics*	
Type of Surgery	
COMMANDO	152 (69.4)
Local excision	67 (30.6)
Neck Dissection	
None	8 (3.7)
Yes, unilateral	154 (70.3)
Yes, bilateral	57 (26.0)
Dose fractionation (tumor bed)	
35x2 Gy (70 Gy)	1 (0.5)
33x2 Gy (66 Gy)	111 (50.7)
30x2 Gy (60 Gy)	58 (26.5)
28x2 Gy (56 Gy)	30 (13.7)
25-26x2.5 Gy (62–65 Gy)	6 (2.7)
22-24x2.5 Gy (55–60 Gy)	2 (0.9)
33x1.8 Gy (59.4 Gy)#	8 (3.7)
No PORT tumor bed	3 (1.4)
Accelerated (6 fractions/week)	103 (47.0)
Neck PORT	
No target	132 (60.3)
Yes, unilateral	64 (29.2)
Yes, bilateral	23 (10.5)
PORT + chemotherapy	17 (7.8)

Abbreviations: COMMANDO=Combined Mandibulectomy and Neck Dissection, PORT = postoperative radiotherapy.

# in combination with a 33x2 Gy dose prescription to nodal areas

At 5-year follow-up, 123 patients were event-free (116 patients with complete follow-up, 1 patient emigrated, 6 patients with follow-up 1–3.6 year), 47 patients had tumor progression, and 65 patients were censored: 12 patients with second primary OSCC, 4 patients with another second primary head & neck tumor, 12 patients had a second primary lung tumor, 8 patients had another second primary tumor, and 13 patients died from non-cancer related causes. With respect to survival, 104 patients died within eight years, and median follow-up of patients alive was eight years. Two patients emigrated, and ten patients were known with a survival < six years with no recent information in the previous 1.5 year on their status in the patient files.

### Tumor progression

**Fig. 2:**
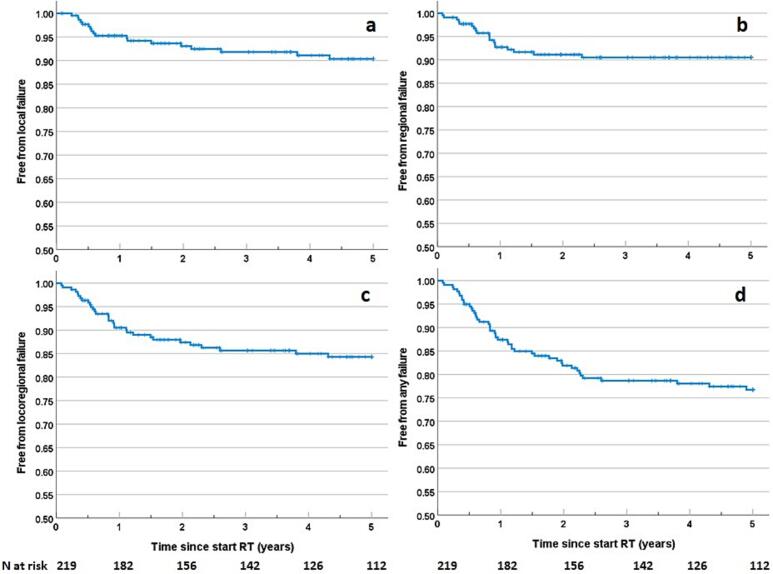
Kaplan Meier plots for the endpoints **a**) local failure, **b**) regional failure, **c**) locoregional failure, and **d**) any failure (local, regional, or distant).

First site of tumor progression within 5-years (n = 47) was: local failure (LF) n = 10, regional failure (RF) n = 10, distant failure (DF) n = 16, LF + RF n = 6, DF + LF n = 2, DF + RF n = 3. Free from any tumor progression rates (1 standard error (1SE)) were: 81.9 % (2.7) at 2y, 78.7 % (2.9) at 3y, and 76.7 % (3.0) at 5y (Fig. 2). One LF was observed in a patient who had only regional PORT. As stated in the Methods section, infield/outfield classification for local failures in patients with PORT to the tumor bed, was not feasible. One patient was known with a LF after 6.5y which was considered as a recurrence and not as a new OSCC by the tumor board (not included in the analyses). Most LRFs occurred within 2.5 years: 29/31 (94 %). RFs were present in 3 out of 23 patients (13.0 %) with bilateral neck RT, 4/64 patients (6.3 %) with lateralized neck RT (all contralateral, outfield), and 12/132 (9.1 %) of N0-1 patients (4/107 N0, 8/25 N1) with no neck RT (n = 8 contralateral after unilateral neck dissection). Detailed patient and recurrence characteristics for all LRF cases are provided in supplementary Table A2.

Locoregional control rates were (1SE): 87.3 % (2.3) at 2-year, 85.7 % (2.5) at 3-year, and 84.3 % (2.6) at 5-year (Fig. 2). Local control rates were (1SE): 93.1 % (1.8) at 2-year, 91.8 % (2.0) at 3-year, and 90.4 % (2.2) at 5-year. Regional control rates were (1SE) 91.1 % (2.0) at 2-year, and 90.5 % (2.1) at 3–5-year.

Salvage treatment with curative intent (after the initial PORT) was performed in seven patients (mainly ND plus neck PORT), and in two LF patients (local excision, subsequent LF afterwards). None of the salvaged RF patients developed LF or RF afterwards but two patients developed metastasis, and one patient died of complications after the surgery. One patient had regional progression during PORT in a non-irradiated area, and had extension of the RT fields and increased dose levels during the RT course; this patient had again RF shortly after completion of the PORT.

### Prognostic factors

**Fig. 3:**
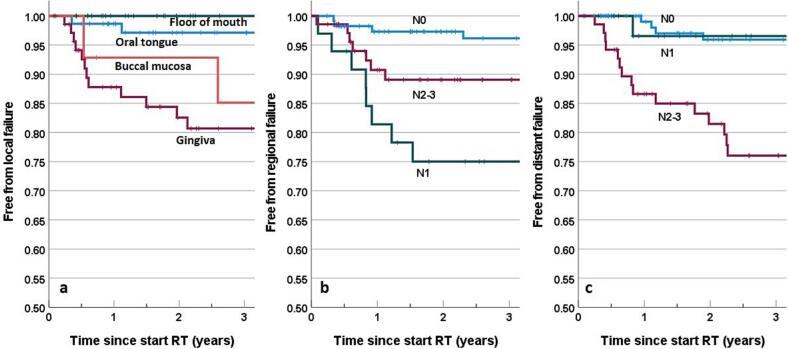
Kaplan Meier plots for the first event of **a**) local failure (tumor site subgroups), **b)** regional failure (N stage subgroups), and **c)** distant failure (N stage subgroups).

Prognostic factors for LF as first site of failure were evaluated within the group receiving tumor bed PORT (N = 216). Significant prognostic factors at univariable analysis were (Table 2): T4 stage (HR = 3.5), bone invasion (HR = 4.0), and tumor subsite gingiva (increased risk with respect to tongue and floor of mouth, HR = 15). The factors T4, bone invasion, and subsite gingiva, all refer to the same patient subgroup (N = 12) with a T4 gingiva tumor with bone invasion that had LF. At multivariable analysis, only tumor subsite gingiva remained significant for LF. For RF, we identified pathological N1 stage (HR = 7.4) as a significant prognostic factor with N0 as reference (Table 3). DF as first site of failure was significantly associated with N stage (p < 0.001): 15 out of 21 patients (71 %) who had DF as first failure, were stage N2-3 (Fig. 3).

**Table 2:** Results of univariable Cox proportional hazards regression for the endpoint of local failure in patients who received PORT on the tumor bed (N = 216 patients, 17 events). Significant results are in bold.

Prognostic Factor	Hazard Ratio	95 % CI	p value
*Patient Factors*			
Age at PORT (≥65 vs < 65 years)	0.93	0.36–2.42	0.9
Sex (female vs male)	1.06	0.40–2.78	0.9
Active smoker at diagnosis (yes vs no)	0.91	0.35–2.36	0.8
*Tumor Factors*			
pT stage (T4 vs T1-3)	**3.47**	1.22–9.85	**0.02**
pN stage (N0 is ref)			0.9
N1	0.75	0.18–3.86	0.7
N2-3	0.94	0.32–2.76	0.9
Bone invasion (yes vs no)	**4.02**	1.49–10.9	**0.006**
Subsite (oral tongue is ref)			**0.008**
Floor of Mouth Gingiva	1.25 **15.29**	0.08–20.0 1.83–117	0.9 **0.009**
Buccal mucosa	9.70	0.88–107	0.064
*Pathology*			
Positive margins or <1 mm (yes vs no)	1.09	0.42–2.87	0.9
Positive margins or <5 mm (yes vs no)	0.99	0.28–3.44	1.0
Budding growth (yes vs no)	0.84	0.31–2.28	0.7
Perineural invasion (yes vs no)	0.70	0.25–1.99	0.5
Lympho-vascular space invasion (yes vs no)	0.97	0.28–3.38	1.0
Differentiation grade (Moderate is reference*)			0.4
Good	1.85	0.41–8.25	0.4
Poor	0.54	0.15–1.92	0.3

Abbreviations: ref = reference category; PORT = postoperative radiotherapy; p = pathological.

* we choose moderate as reference category because only n = 11 patients had good differentiation.

**Table 3:** Results of univariable Cox proportional hazards modeling for the endpoint of regional failure (N = 19 events). Significant results are in bold.

Prognostic Factor	Hazard Ratio	95 % CI	p value
*Patient Factors*			
Age at start of PORT (≥65 vs < 65 years)	0.49	0.19–1.23	0.13
Sex (female vs male)	0.41	0.14–1.25	0.12
Active smoker (yes vs no)	1.73	0.68–4.39	0.25
*Tumor Factors*			
Pathological T-stage (T4 vs T1-3)	0.67	0.26–1.77	0.4
Pathological N-stage (N0 is ref)			**0.003**
N1	**7.37**	2.22–24.47	**0.001**
N2-3	3.08	0.90–10.53	0.073
Bone invasion (yes vs no)	1.01	0.38–2.66	0.9
Budding growth (yes vs no)	1.29	0.46–3.58	0.6
Perineural invasion (yes vs no)	0.95	0.37–2.41	0.9
Lymphovascular space invasion (yes vs no)	2.18	0.83–5.73	0.12
Differentiation grade (Moderate is ref*)			0.9
Good	0.92	0.121–7.049	0.9
Poor	0.82	0.29–2.29	0.8
Tumor subsite (oral tongue is ref)			0.19
Floor of mouth	0.49	0.13–1.84	0.3
Gingiva	1.23	0.46–3.28	0.7
Buccal mucosa	0 (no events)	−	−

Abbreviations: ref = reference category; PORT = postoperative radiotherapy.

* we choose moderate as reference category because only n = 12 patients had good differentiation.

### Survival

Overall survival (OS) at 5- and 8-year (1SE) was 63.0 % (3.3) and 48.3 % (3.7), respectively (supplementary Fig. A1). Cause of death was: index OSCC tumor (n = 42), second OSCC tumor (n = 9), other head & neck tumor (n = 3), lung cancer (n = 12), other cancer (n = 13), severe illness/poor condition partly related to chronic toxicity (n = 6, e.g. dysphagia, anorexia, aspiration pneumonitis, trismus, and/or osteoradionecrosis), other non-cancer-related causes (n = 19, e.g. cardiovascular events, severe COPD), and in one case it was unknown. Significant prognostic factors at multivariable analysis were the Charlson Comorbidity Index Score (CCI) (HR = 1.7 for score ≥ 2 versus score 0), N stage (N2-3 vs N0, HR = 2.4), and tumor subsite gingiva (HR = 2.4 vs oral tongue), supplementary Table A3.

## Discussion

We performed a retrospective cohort study in high-risk OSCC patients treated with PORT. At 5-years, a locoregional control rate (LCR) of 84 % was achieved after selective node dissection and PORT, with only 8 % receiving concomitant chemotherapy. Freedom from any tumor progression was 77 %. LF as a first event was observed in 18 cases, with most cases concerning a T4 gingiva tumor with bone invasion (n = 12). RF as first event was observed in 19 patients of which 10 cases concerned solitary RFs mainly situated in contralateral neck regions with no PORT indication. OS at 5-year and 8-year was 63 % and 48 %, respectively. The index tumor and subsequent new primary tumors during follow-up had both a substantial contribution to the death rate.

Comparing our outcomes with other studies concerning PORT for OSCC is challenging, since studies on this topic are heterogeneous with respect to baseline characteristics (e.g. tumor subsites), PORT indication, follow-up, and indications for concomitant chemotherapy. Quinlan-Davison et al evaluated a comparable patient group (with n = 289) and reported a similar 5-year LCR of 76 % and OS of 57 % [13]. In general, studies evaluating OSCC patients receiving PORT +/- chemotherapy with modern RT, reported 2-3y LCR rates in the range of 75 %-90 % [[13], [14], [15],[20], [21], [22], [23], [24]]. Most studies prescribed comparable dose levels in the range of 54–70 Gy to the target volumes, using advanced RT techniques.

In our study, we observed four contralateral RFs in the subgroup receiving lateralized (ipsilateral) neck PORT. This rate of 6.3 % is similar to the average rate of 5.9 % that was reported in the *meta*-analysis of Razavian et al [27]. All four cases concerned oral tongue tumors with N stage pN2b. In the group without neck RT indication we observed mainly contralateral cases, and a remarkable high RF rate of 32 % for N1 cases (8 out of 25). Risks of regional spread in (untreated) neck areas has been a topic of debate [23,25,26]. A diverted lymphatic drainage was identified as an explanatory factor for contralateral RFs [23,25]. The observed high RF rate in the N1 group is not in agreement with literature and possibly a statistical outlier. Geretschläger et al, and Mione et al observed mainly RF cases in PORT areas [20,21]. Yosefof et al reported that all identified contralateral RFs were in neck areas without PORT indication and without ND during the primary surgery, because no contralateral nodes were noticed clinically or on imaging, similar to our observations [23].

We identified tumor subsite, T stage, N stage, and bone invasion as prognostic factors for LRF, with highest LF rates for subsite gingiva. Other studies have identified additional prognostic factors for LRF or disease-free survival, like differentiation grade, ENE, lympho-vascular space invasion, depth of invasion, nodal ratio, positive or close margins, age, and tumor dose [7,[13], [14], [15],[20], [21], [22], [23], [24], [25], [26]]. Depth of invasion has more recently been proposed as a relevant prognostic factor in low-risk OSCC to identify subgroups possibly requiring additional treatment, and was not evaluated in the current high-risk study population [27].

Limitations of the current study is its retrospective nature, and the lack of sufficient local recurrence imaging for in/outfield classification. Strengths are its reasonably large sample size, the completeness of 5-year follow-up at the outpatient clinic for the majority of patients, and the available detailed information from the radiotherapy systems on the planned and delivered treatment. Furthermore, the selected PORT patient population may differ to some extent from current clinical practice because guidelines for selection and treatment have changed over the last ten years.

In conclusion, we established that the obtained tumor control and OS in our cohort was comparable to literature. The risk of LF was highest for T4 gingiva tumors with bone invasion. We observed a high risk of RF in N1 cases in contralateral neck areas without PORT indication and without initial neck dissection, which was not in agreement with literature. The risk of distant failure as a first event was highest in N2-3 cases, associated with worse OS. Cancer-related death was the dominant cause of death in this population, related to both the index cancer and other cancers. The results of the current study with a relatively large sample size and complete follow-up may serve as a benchmark for future evaluations of evolving clinical protocols in high-risk OSCC patients receiving PORT.

## Funding statement

This research did not receive funding by external parties.

## Declaration of competing interest

The authors declare the following financial interests/personal relationships which may be considered as potential competing interests: [Insert text provided in the DoCI form].
