# Supplementary material for: Tumor control and survival after postoperative radiotherapy for high-risk oral cavity cancer: A retrospective cohort study
Source: Clin Transl Radiat Oncol. 2025 May 30;53:100988. doi: 10.1016/j.ctro.2025.100988 (PMC12166991; doi:10.1016/j.ctro.2025.100988)

Appendix: Supplementary Files

Manuscript: Tumor control and survival after postoperative radiotherapy for high-risk oral cavity cancer: a retrospective cohort study. Bolleurs et al.

Supplementary Table A1. Distribution of pathological risk criteria for PORT indication, per tumor subsite.

Risk factor	Oral tongue	Floor of Mouth	Gingiva	Buccal Mucosa
pT4	12%	32%	87%	36%
Budding pattern	74%	65%	61%	79%
pN2-3	39%	33%	21%	43%
PNI	54%	43%	24%	21%
Lymphovascular invasion	23%	18%	10%	29%
Positive margins	8%	45%	41%	21%
Positive or < 1mm	16%	58%	48%	50%
Positive or <5mm	70%	93%	83%	93%

Abbreviations: PNI = perineural invasion

Supplementary Table A2. Characteristics of n=31 patients with local and/or regional failure.

**N**	**Tumor subsite**	**pTN stage**	**Dose Tumor bed**	**Neck RT**	**ND**	**Time to failure**	**Location of recurrence description**	**Type of failure**
**1**	Right side floor of mouth and oral tongue	T2N1	28x2 Gy	None	Right	15 mo	Left neck LN levels II and III	RF CL rN2c
**2**	Left side oral tongue	T3N1	30x2 Gy	None	Left	11 mo	Right neck LN level I	RF CL rN2a
**3**	Left edge oral tongue and floor of mouth	T3N2B	33x2 Gy	Left	Left	10 mo	Right neck, submandibl location, and distant metas	RF CL rN2b
**4**	Left edge of oral tongue	T2N0	33x2 Gy	None	Left	11 mo	Right neck LN level III and IV	RF CL
**5**	Left gum of mandible (trigonum retromol)	T3N1	33x2 Gy	None	Left	10 mo	Right neck LN level II, and distant metastasis	RF CL rN2c
**6**	Left gum maxilla (proc alveolaris)	T4aN0	33x2 Gy	None	Left	28 mo	Unknown side, neck and submandibular location	RF rN1
**7**	Left side floor of mouth	T1N1	33x2 Gy	None	Left	1 mo	Right neck LN level unknown	RF CL rN2c
**8**	Right front 2/3 oral tongue	T2N2b	none	Right	Right	11 mo	Left neck LN levels II and III	RF CL rN3
**9**	Right side of oral tongue	T2N2b	30x2 Gy	Right	Right	8 mo	Left neck LN levels I and II + (sub)cutaneous metas	RF CL
**10**	Right posterior edge oral tongue	T1N1	30x2 Gy	None	Right	18 mo	Left neck LN level III	RF CL rN2c
**11**	Right gum mandible (proc alveolaris)	T4aN1	33x2 Gy	None	Right	4 mo	Left neck LN level III proven, Level I and LN supraclavicular suspect (no punction)	RF CL rN2b
**12**	Central floor of mouth	T1N1	30x2 Gy	None	L+R	10 mo	Left neck LN level unknown	RF rN1
**13**	Gum mandibular (process alveolaris)	T4N2c	33x2 Gy	L+R	L+R	1 mo	Left+right supraclavicular	RF rN2c
**14**	Central gum maxilla (proc alveolaris)	T4aN0	30x2 Gy	None	L+R	24 mo	Left maxillary sinus and infra-orbital nerve	LF
**15**	Left gum mandible (proc alveolaris)	T4aN0	28x2 Gy	None	Left	5 mo	Left gum of mandible	LF rTis
**16**	Left gum mandible (proc alveolaris)	T4aN0	30x2 Gy	None	Left	18 mo	Right, paramedian gum of mandible	LF rT2
**17**	Left buccal mucosa	T1N1	33x2 Gy	Left	Left	6 mo	Left gum maxilla + left buccal mucosa	LF rT4a
**18**	Right buccal mucosa	T2N2b	30x2 Gy	Right	Right	31 mo	Buccal mucosa, right	LF rT4
**19**	Right gum mandible (proc alveolaris)	T4aN2 b	33x2 Gy	Right	Right	5 mo	Right gum	LF rT4
**20**	Right gum mandible (trigonum retromol)	T4aN0	33x2 Gy	None	Right	6 mo	Right gum mandible	LF rT4b
**21**	Right gum mandible (proc alveolaris)	T4bN0	33x2 Gy	None	Right	26 mo	Buccal mucosa right	LF rT4a
**22**	Right gum mandible (proc alveolaris)	T4aN0	34x2 Gy	None	Right	52 mo	Right edge of oral tongue	LF rT2
**23**	Right gum mandible (proc alveolaris)	T4aN0	28x2 Gy	None	L+R	3 mo	Left mandible	LF rT4a
**24**	Right floor mouth	T2N2b	33x2 Gy	Right	L+R	46 mo	Right edge of oral tongue	LF rT2
**25**	Right gum mandible (proc alveolaris)	T4aN0	30x2 Gy	None	L+R	13 mo	Local recurrence submandibularis	LF rTx
**26**	Right gum maxilla (proc alveolaris)	T4aN0	33x2 Gy	None	None	4 mo	Tumor mass right- buccal space, masti-cator loge, masseter + LN level II right	LF+RF IL rT4N2a

**27**	Right side oral tongue and floor of mouth	T3N0	28x2 Gy	None	Right	4 mo	Region removed submandibular gland + Left neck LN level I	LF+RF CL rTxN2c
**28**	Right edge oral tongue	T2N2b	none	Right	Right	13 mo	Floor of mouth with submental and cutaneous extension + LN level III/IV left	LF+RF CL rT4aN1
**29**	Left gum of mandible (proc alveolaris)	T4aN2c	33x2 Gy	L+R	L+R	7 mo	Right growth in sternocleido-mastoid muscle + LN level III right	LF+RF CL rT4N3
**30**	Left gum mandible (proc alveolaris)	T4bN1	28x2 Gy	None	Left	7 mo	Left growth in masticator space + LN levels II and V left	LF+RF IL
**31**	Right gum mandible (proc alveolaris)	T2N2b	33x2 Gy	L+R	L+R	7 mo	Tumor mass, not original location but suspected growth based on perineural extension + LNs in parotid gland right	LF+RF IL rT4N2b

Abbreviations: mo=months; LF=local failure; RF=regional failure; L+R=left+right; ND=neck dissection; LN=lymph node; RT=radiotherapy; CL=contralateral; IL=ipsilateral.

Supplementary Table A3. Results of Cox proportional hazards modeling for overall survival (N=104 events). All analyses were evaluated in a baseline model with adjustment for age at start PORT. Significant results are in bold.

Prognostic Factor	Hazard Ratio	95% CI	p value
Patient Factors			
Age at start of PORT (per 10 year increase)	1.16	0.98-1.38	0.088
Sex (female vs male)	0.73	0.49-1.10	0.14
Active smoker (yes vs no)	1.38	0.93-2.05	0.11
Charlson Comorbidity score# (0 is ref)			0.066
1	1.40	0.86-2.29	0.18
≥2	**1.71**	1.08-2.69	**0.02**
Tumor Factors			
Pathological T-stage (T4 vs T1-3)	**1.48**	1.01-2.18	**0.045**
Pathological N-stage (N0 is ref)			**0.010**
N1	1.11	0.61-2.04	0.8
N2-3	**1.88**	1.24-2.85	**0.003**
Tumor subsite (oral tongue is ref)			0.11
Floor of mouth	1.29	0.76-2.17	0.3
Gingiva	**1.77**	1.09-2.87	**0.02**
Buccal mucosa	0.96	0.40-2.32	0.9

Abbreviations: ref = reference category; PORT=postoperative radiotherapy

without age correction, the Hazard Ratio=1.46 (p=0.13) and 1.80 (p=0.01), respectively.

Supplementary Figure A1. Kaplan Meier plot for overall survival**.**

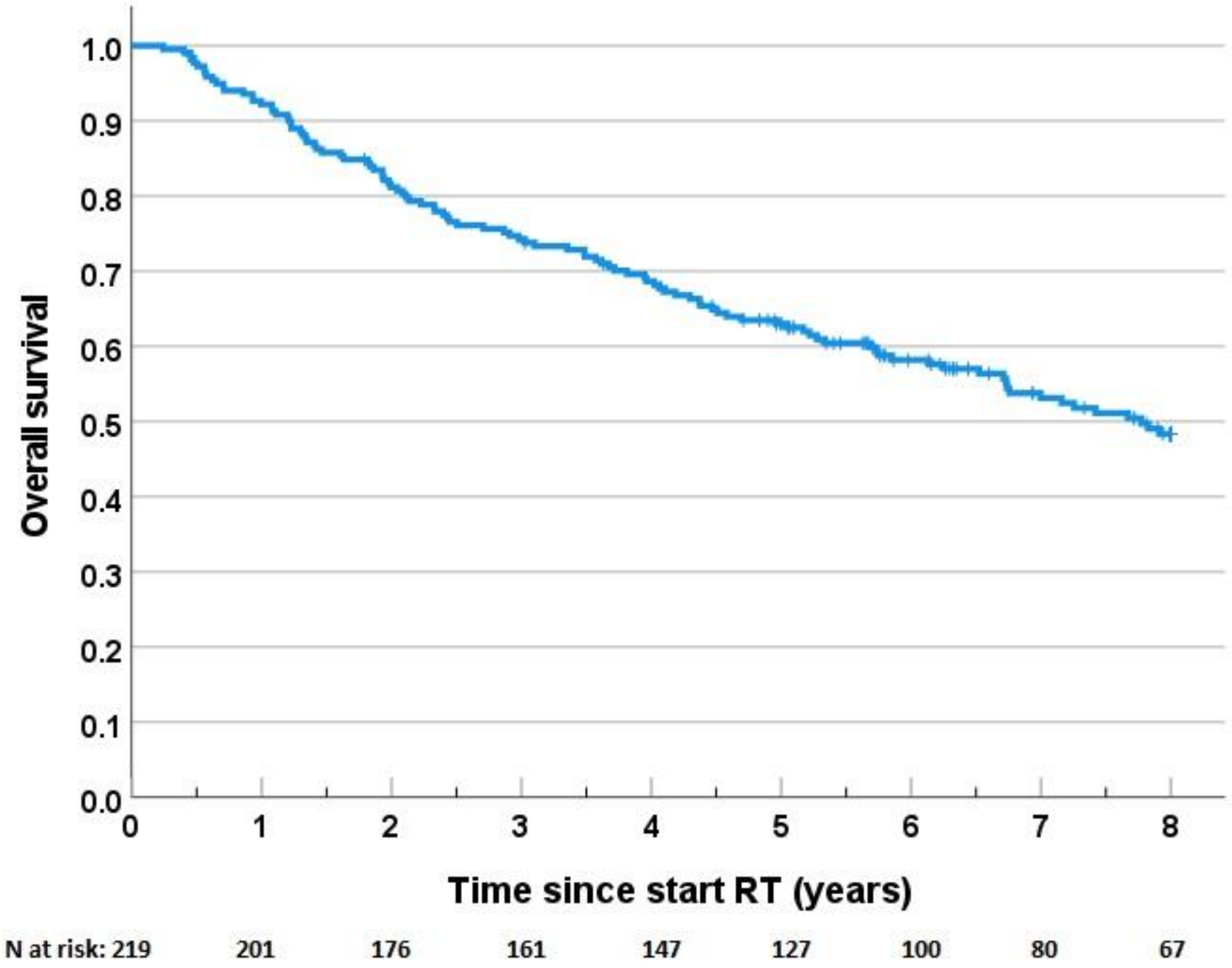

Supplement: Supplementary Data 1 [file mmc1.pdf]
